# Antimalarial Efficacy and Toxicological Assessment of Extracts of Some Ghanaian Medicinal Plants

**DOI:** 10.1155/2019/1630405

**Published:** 2019-08-01

**Authors:** Michael Konney Laryea, Lawrence Sheringham Borquaye

**Affiliations:** ^1^Department of Chemistry, Kwame Nkrumah University of Science and Technology, Kumasi, Ghana; ^2^Central Laboratory, Kwame Nkrumah University of Science and Technology, Kumasi, Ghana

## Abstract

The economic costs associated with morbidity and mortality due to malaria and malaria associated complications in many sub-Saharan countries and other malaria endemic regions of the world are huge. Reports of emergence of parasite resistance to current malaria drugs have complicated malaria treatment and require the development of new therapeutic agents. The folkloric use of medicinal plants for the management of malaria is well documented. This work evaluated the antiplasmodial activities and toxicity of some medicinal plants used to treat malaria and malaria-like symptoms in Ghana. Plant extracts were obtained by cold maceration in 70% ethanol. Antiplasmodial efficacies were assessed* in vitro* against 3 strains of* Plasmodium falciparum* strains (FCM, W2, and CAM06) and* in vivo* via the 4-day suppressive test in* Plasmodium berghei* infected mice. Cytotoxicity and acute toxicity were assessed in mammalian cells and mice, respectively. All extracts were active against at least one of the* Plasmodium falciparum* strains in* in vitro* evaluations with IC_50_'s in the range of 4–116 *μ*g/mL, whereas* Bidens pilosa* extracts, with a chemosuppression rate of 75%, was the most active plant in the* in vivo* experiments. All plant extracts displayed very weak to no cytotoxicity against the mammalian cell line used and exhibited very good selectivity towards the* Plasmodium* parasites.* Syzygium guineense* and* Parinari congensis* extracts were the most toxic in the acute toxicity tests. Altogether, the results indicate that the medicinal plants do possess impressive antiplasmodial properties and provide scientific basis for their use in traditional herbal medicine.

## 1. Introduction

Malaria is one of the major public health challenges in many countries all over the world. Over 200 million malaria cases are reported every year with more than 420,000 deaths as a result of malaria recorded annually. The malaria disease is endemic in over 90 countries, which implies about 40% of the world's population are either affected or at risk. Most malaria morbidity and mortality are reported from sub-Saharan Africa. Ninety percent of all reported cases and deaths are from the region. In the Democratic Republic of Congo and Angola, about 34,000 and 16,000 deaths, respectively, were attributed to malaria in 2016. Reported deaths in Ghana due to malaria were estimated at about 1,300. In sub-Saharan Africa, majority of malaria victims are pregnant women and children under 5 years [1].

The United Nations (UN), as part of its Millennium Development Goals (MDGs), included a specific objective to address the challenges posed by malaria. This, amongst others, led to unprecedented declines in malaria morbidity and mortality in the past decade. Malaria cases and deaths dropped by more than 50% and millions of lives were saved with antimalarial drugs and vector control initiatives [2]. In recent years, however, there has been a slow decline in the reduction rates of malaria infection. Recent reports from the World Health Organization (WHO) has revealed that the fight against malaria with available tools and funding is now at crossroads and this leaves many children and pregnant women at risk of getting the infection [1, 3].

One key challenge facing the fight against the disease is the phenomenon of drug resistance. Effective malaria control and eradication depend largely on treatment with efficacious antimalarial drugs. Antimalarial drugs such as quinine, chloroquine, and artemisinin and its derivatives have over the past years been the gold standard for treating malaria. Drug resistance, however, has led to inefficacy or reduced efficacy of these drugs [4, 5]. Despite the enormous success of artemisinin and its derivatives, in combination therapies as described by the WHO, recent reports of* Plasmodium* resistance in South East Asia raises an eyebrow [6, 7]. An urgent need for new and improved antimalarial therapeutics, preferably with novel routes of action to prevent, control, or minimize parasite resistance, is needed in the drug discovery pipeline. This is necessary in the event that artemisinin-resistant* Plasmodium* parasites become widespread throughout the world.

Indigenous use of plants as health restorative products has been around for centuries. It has been validated that remedies prepared from plants have the capacity to combat several types of diseases [8]. According to the WHO, about 70% of the world's population rely on herbal preparations for the treatment of diseases [9, 10]. In Ghana, herbal remedies are utilized by about 60 to 70% of the population in the rural areas [11] and increasingly, the urban population is also using them [12]. The use of traditional remedies provides a cheaper, easier, and sustainable alternative to most synthetic drugs and pharmaceuticals. Additionally, they are perceived to rarely produce any side effects and are tolerated with fewer unintended consequences [13]. It has been estimated that, of the over 1,000 medicinal plants in Ghana, about 40% are used in the treatment of various diseases such as malaria, asthma, jaundice, typhoid fever, diabetes, hypertension, and anemia [14, 15]. In Ghana, 6% of medicinal plants on the domestic market are used in malaria and fever management [15]. Many of these plants have been formulated as registered commercial products and are available to the public as malaria therapeutic agents. A key challenge in the use of medicinal plants in malaria treatment is the absence of scientific evidence of their efficacies. Additionally, toxicological profiles of these plants are either missing or scanty at best. In this study, 11 medicinal plants that are ethnomedically used in the treatment of malaria and malaria-like symptoms in Ghana were investigated for their antiplasmodial activities and toxicity profiles. Crude ethanolic extracts from these plants was tested* in vitro* against three different strains of the* Plasmodium* parasite:* Plasmodium falciparum* chloroquine-sensitive (FCB),* Plasmodium falciparum* chloroquine-resistant (W2), and a field isolate (coded as CAM06, obtained from a local patient).* In vivo* antiplasmodial activity (4-day suppressive tests) was evaluated in* Plasmodium berghei* infected BALB/C mice. The ethanolic extracts were also assessed for cytotoxicity (against LLC-MK2 monkey kidney epithelial cells) and acute toxicity (in mice).

## 2. Methods

### 2.1. Collection of Plant Material

Eleven plants ethnomedically used for the treatment of malaria and malaria-like symptoms in Ghana were selected for this work. The plant sample was collected from various parts of Ghana between August and December 2017 and was identified and authenticated by Mr. Clifford Asare, a plant botanist at the Department of Herbal Medicine, Faculty of Pharmacy and Pharmaceutical Sciences at the Kwame Nkrumah University of Science and Technology (KNUST). Specimens with voucher numbers were deposited in the herbarium. Harvested plant materials were washed under running water to remover foreign matter. Plant materials were air-dried at ambient temperatures under a shade for up to 2 weeks, depending on plant part, and pulverized into course powders.  Table 1 describes the parts of the various plants used for the study and their local indications.

### 2.2. Extraction

One hundred grams of each powdered plant material was macerated in 70% ethanol for a minimum of 5 days. The residue was separated from the menstruum via filtration and concentrated* in vacuo* (Cole Parmer Rotary Evaporator N-1110, China). They were then transferred into screw-capped vials and stored at 4°C until use.

### 2.3. Phytochemical Analysis

Plant extracts were tested for the presence of various phytochemicals: tannins, steroids, flavonoids, alkaloids, coumarins, and glycosides. The tests were done following standard procedures [18–21].

### 2.4. *In Vitro* Antiplasmodial Activity

The plant extracts were assayed against* Plasmodium falciparum* chloroquine-sensitive (FCB),* P. falciparum* chloroquine-resistant (W2), and a* P. falciparum* field isolate (coded CAM06, obtained from local patients) strains using the lactate dehydrogenase (pLDH) method previously described by [22] and modified by [23, 24]. Parasites were cultured* in vitro* according to the method of [25]. Parasites were grown in uninfected O^+^ human red blood cells as host cells and maintained in RPMI 1640 containing 2.5% hematocrit, hypoxanthine (0.5 × 100^−2^% w/v), HEPES (59.4 × 100^−2^% w/v), glucose (0.25 × 100^−2^% w/v), and albuMAX II (50.0 × 100^−2^% w/v), and buffered with NaHCO_3_ to a pH of 7.34. Fresh culture was maintained for at least 96 hours (2 complete life cycles) before being used for assays. All solutions were filter-sterilized with 0.22 *μ*m syringe-adapted filters (Corning®, NY, USA). Assays were performed in 96-well culture plates and all IC_50_ values were obtained from dose-response curves.

### 2.5. *In Vitro* Cytotoxicity

Cytotoxicity was evaluated on LLC-MK2 monkey kidney epithelial cells. Cells were grown in DMEM culture medium supplemented with 10% fecal bovine serum (FBS, Life Technologies) and 1% penicillin/streptomycin. Trypsinated cells were distributed in 96-well microtiter plates at 10,000 cells in 100 *μ*L per well and incubated for 48 hours to allow them to attach before adding the extracts. After 48 hours, the medium was removed completely from each well, and 100 *μ*L of fresh culture medium was added. Thereafter, 100 *μ*L of crude extracts (400 *μ*g/mL) was added and then serially diluted. Cells without drug/extract addition (100% growth) served as controls. The cells with or without extracts were incubated at 37°C in 5% CO_2_, 5% O_2_, and 90% N_2_ incubator for 72 h before determining their viability. Each concentration was tested in triplicate. Cell viability was determined by MTT assay and the cytotoxic activity was determined as described elsewhere [24, 26]. The percentage viability and percentage mortality were calculated from the OD values using Microsoft Excel. The mean results of the percentage mortality were plotted against the logarithms of concentrations using GraphPad Prism. Regression equations obtained from the graphs were used to calculate cytotoxic concentration fifty (CC_50_), which is the concentration of extract required to kill fifty percent of the cells. The selective index (SI) was computed as the ratio of CC_50_ to IC_50_ value.

### 2.6. *In Vivo* Acute Toxicity

This was done through assessment of the acute toxicity of the extracts, using male Balb/C mice, according to Organization for Economic Cooperation and Development (OECD) guidelines for testing of chemicals acute oral toxicity [27]. Healthy young adult male mice aged 8-10 weeks (18-30 g body weight) were used. In brief, for each extract, animals were randomly divided into groups of 5 animals each. A control group was maintained without any treatment (receiving only the vehicle). The animals in all the experimental groups were kept in their respective cages for 5 days for acclimatization prior to the start of the experiment. Animals in the eleven test groups received plant extract at a fixed dose of 5000 mg/kg, while those of the control group were administered the vehicle (distilled water). Animals were fasted 4 hours prior to dosing and 2 hours after dosing with extract or vehicle, while water was made available throughout the experiment. Apart from these exceptions, the animals also received feed* ad libitum* throughout the experiment. The mice were observed periodically for 4 hours following administration of extract/vehicle and every day for 14 days for detection of mortality as well as any behavioral alterations. Feed and water consumption were monitored. Body weight was also recorded on days 7 and 14 [28].

### 2.7. *In Vivo* Antiplasmodial Activity

The* in vivo* antiplasmodial activities of the extracts with significant* in vitro* antiplasmodial activity were evaluated via the 4-day suppressive test against* Plasmodium berghei* infections in mice. Extracts were prepared by dissolving 5 mg dry crude extracts in 200 *μ*L DMSO (Sigma, MO, USA) and then diluted with distilled water to the desired concentration.

#### 2.7.1. Animal and Parasite

BALB/C mice (Male and Female) of age 6–8 weeks and weight 27–32 g were maintained at a temperature of 25°C and a 12-hour light/12-hour dark cycle, with food and water given* ad libitum* in the Animal House of MRABL at the Faculty of Health Sciences, University of Buea, Cameroon. All experiments were conducted in accordance with internationally accepted laboratory animal use.

#### 2.7.2. Parasite Inoculation

Donor albino mice previously infected with* Plasmodium berghei* and having parasitemia level of 20-30% were used. The donor mice were anesthetized and sacrificed by opening the thoracic region in order to expose the heart. Blood was collected by cardiac puncture into heparinized vacutainer tube containing 0.5% trisodium citrate. Physiological saline (0.9%) was used to dilute the blood based on the parasitemia level of the donor mice [28, 29]

#### 2.7.3. Suppressive Test

The 4-day suppressive test was used to evaluate the* in vivo* schizonticidal activity of three of the plants (*Bidens pilosa*,* Paspalum scrobiculatum,* and* Clappertonia ficifolia*) against* Plasmodium berghei* infected BALB/C mice following reported methods [28, 30] with some modifications. Briefly, infected mice were randomly divided into four groups of 5 each by weight. Treatment started three hours after mice have been inoculated with parasite on day 0 and continued for four days (day zero inclusive). For each extract, animals received daily oral dose of 400 mg kg^−1^ day^−1^ in 100 *μ*L vehicle (2% DMSO in distilled water). A positive control-group received 10 mg/kg weight quinine per day, while the negative-control group animals were administered 100 *μ*L of the vehicle. On the 4th day after treatment, blood sample was obtained from the tail of each mouse. A thin film was prepared and stained with Giemsa to determine parasitemia levels. The mean parasitemia in each group was determined and the % chemosuppression of each sample was computed as [(*A* –* B*)/*A*] x 100, where *A* was the mean parasitemia in the negative control group and *B* was the mean parasitemia in the test group.

## 3. Results

In this study, the antiplasmodial activities of 11 medicinal plants commonly used in Ghana for malaria treatment were evaluated in both* in vitro* and* in vivo* assays. Additionally, the cytotoxicity of the plant extracts towards mammalian cell lines and acute toxicity in mice models were also assessed. The 11 plants chosen for this study were obtained from informal discussions with local traditional healers, indigenes, and literature search. The 11 plants belonged to different families and are used in treating ailments such as fever, malaria, stomach aches, and infections. Plant extracts were obtained by cold maceration in 70% ethanol. The yields of the extraction ranged from 5.0 to 15.0% (Table 1).

Phytochemical screening for the major chemical classes revealed the presence of tannins, steroids, flavonoids, alkaloids, coumarins, and glycosides which may be responsible for the observed antiplasmodial activities, as shown in Table 2. The ubiquitous phytochemical, tannin, was present in all plant samples investigated in this study, whereas alkaloids were absent in all plants except* Faurea speciosa*. Steroids, flavonoids, coumarins, and glycosides were present in at least 5 plant samples. None of the plants studied contained all six phytochemicals tested for. In* Paspalum scrobiculatum*, the only phytochemical present was tannins.

To determine the* in vitro* antiplasmodial activity, plant extracts were screened against the schizont stage of 3 different strains of the* Plasmodium falciparum* parasite: chloroquine-sensitive (FCB), chloroquine-resistant (W2), and CAM06, a field isolate.  Table 3 shows the IC_50_ of the extracts against the 3* Plasmodium falciparum* strains. The guidelines of Jonville et al. [16] that classify antiplasmodial activity as high (IC_50_ <5 *μ*g/mL), promising (5 < IC_50_ <15 *μ*g/mL), moderate (15 < IC_50_ < 50 *μ*g/mL), and inactive (IC_50_ > 50 *μ*g/mL) were adopted for classification of the potency of the various extracts. At low concentrations, inhibition of parasite indicates selective activity, while action at high concentrations may be as a result of nonspecific toxicity. When tested against the chloroquine sensitive FCB strain,* Clappertonia ficifolia* (4.43 ± 0.18 *μ*g/mL) was the only extract that exhibited high antiplasmodial activity. Six other extracts showed promising activities with another 3 showing moderate activity.* Acridocarpus alternifolius*, with an IC_50_ of 175.89 ± 0.37 *μ*g/mL, was inactive. Against the chloroquine-resistant W2 strain,* Bidens pilosa* (4.60 ± 0.91 *μ*g/mL) and* Syzygium guineense* (4.62 ± 1.14 *μ*g/mL) were the most active extracts.* Clappertonia ficifolia* exhibited promising antiplasmodial activity against W2, with an IC_50_ of 7.94 ± 1.36 *μ*g/mL. None of the extracts exhibited high activity towards CAM06.* Clappertonia ficifolia, Faurea speciosa, Syzygium guineense,* and* Croton penduliflorus,* however, had promising activities. Against all strains tested,* Clappertonia ficifolia* was most potent, with IC_50_'s less than 10 *μ*g/mL in all cases.* Faurea speciosa* and* Syzygium guineense* had IC_50_'s less than 15 *μ*g/mL and were also very active.

Following the* in vitro* antiplasmodial tests, the extracts were screened against a mammalian cell line (LLC-MK2 monkey kidney epithelial cells) to evaluate cytotoxicity. The extract concentration that was cytotoxic to 50% of the cells (CC_50_) ranged from 77.9 to >1,000 *μ*g/mL (Table 4). The most cytotoxic extract was* Syzygium guineense* with a CC_50_ of 77.9 ± 0.71 *μ*g/mL. Extracts of* Acridocarpus alternifolius* and* Datura stramonium* were the least toxic to the mammalian cells, with CC_50_ values above 1,000 *μ*g/mL. The selectivity index (SI) of* Paspalum scrobiculatum, Bidens pilosa,* and* Syzygium guineense* extracts towards FCB strain of* P. falciparum* were all < 10 indicating minimal selectivity (Table 4).* Datura stramonium* extract had a much higher selectivity towards the FCB strain relative to the mammalian cell lines used.* Parinari congensis* and* Monanthotaxis caffra* had SI values < 10 for both CAM06 and W2 strains.* Paspalum scrobiculatum* and* Bidens pilosa* also had SI values < 10 for the field strain CAM06.

**Figure 1:**
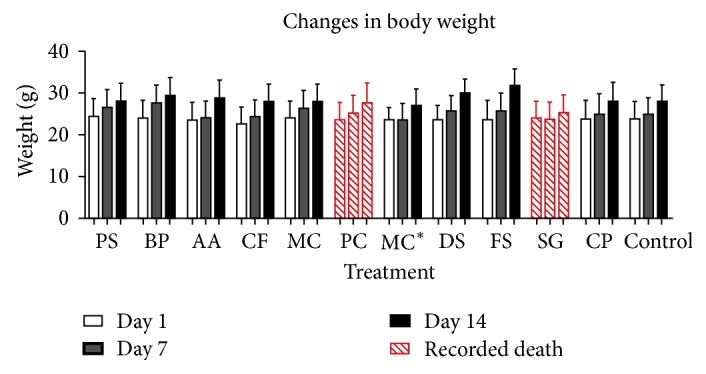
*Acute toxicity: changes in body weight of mice (n=5) upon administration of plant extract over a 2-week period*. PS:* Paspalum scrobiculatum*, BP:* Bidens pilosa*, AA:* Acridocarpus alternifolius*, CF: Clappertonia ficifolia, MC: Mitragyna ciliata, PC:* Parinari congensis*, MC*∗*:* Monanthotaxis caffra*, DS:* Datura stramonium*, FS:* Faurea speciosa*, SG:* Syzygium guineense*, and CP:* Croton penduliflorus*.

In the acute toxicity test, 5000 mg of extract per kg body weight of mice was administered. Upon administration, one animal dead (representing 20%) was recorded in* Syzygium guineense* and* Parinari congensis* during the first 16 hours and 24 hours, respectively. In addition, animals in these two groups were very sluggish at the beginning of the observation period and recovered significantly only after 24 hours. The growth pattern of mice treated with control or extracts were very similar. In virtually all the groups, a weight increase was observed between day 1 and day 14.  Figure 1 illustrates the changes in weight of the various treatment groups over the 2-week period.

Three of the extracts that showed appreciable antiplasmodial activities* in vitro* and are commonly used in Ghana (i.e., have significant traditional importance) were evaluated for* in vivo* antiplasmodial activity using the 4-day suppressive test and the results are summarized in Table 5. There was substantial decrease in percentage parasitemia for all extract treatment groups in comparison to the negative control. Maximal suppression of parasitemia was observed in* Bidens pilosa* treatment groups with a percent chemosuppression of 74.73. All extracts were however inferior to quinine, which recorded a chemosuppression of 90.22%.

## 4. Discussions

Plant medicines have been a source of novel therapeutic agents for various ailments. Two of the most important antimalarial drugs, artemisinin and quinine, originated from* Artemisia annua* and* Cinchona officinalis,* respectively [31]. Plant medicines also play a critical role in primary health care for most of the world's population, especially in malaria treatment [10]. Unfortunately, scientific validation for the use of most of these medicines is absent and represents a major challenge in the industry [32]. This study evaluated the antiplasmodial potential of 11 medicinal plants used for the treatment of malaria and malaria-like symptoms in Ghana. A number of chemical classes of plant origin have been shown to be potential antiplasmodial agents. Alkaloids, terpenes, flavonoids, coumarins, and limonoids are some examples [31, 33]. Phytochemicals may act alone or in synergy with other phytoconstituents to elicit the observed biological activities. It has been reported that alkaloids, flavonoids, and sesquiterpenes are, in general, potent secondary metabolites of plants that display broad-spectrum biological activities [34]. The extracts investigated in this study possessed some of these phytochemicals and may be the reason for the observed antiplasmodial activities.

Of the plants selected for this study, the antiplasmodial activity of 3 plants is present in the literature. The antiplasmodial activities of* Bidens pilosa* have been reported by researchers in Africa and Brazil. A research team in Brazil [35] reported that 50 *μ*g/mL of the ethanol and chloroform extracts of the leaf and stem parts of* Bidens pilosa* inhibited* P. falciparum* growth by 90%. Similarly, [36] reported an inhibition of parasite growth by 45% with ethyl acetate extracts of the same plant. In 2004, Clarkson and coworkers reported an IC_50_ of 5 *μ*g/mL and 70 *μ*g/mL for methanol and water extracts, respectively [37]. In our study,* Bidens pilosa* extracts showed very high activity against the chloroquine-resistant W2 strain, in agreement with earlier reports. Moderate activity was however observed towards the chloroquine-sensitive (FCB) and field strains (CAM06) of* P. falciparum*. The antiplasmodial activities of the root parts of* Bidens pilosa* have been attributed to the presence of polyacetylene and flavonoids [38]. The leaf extracts used in this study tested positive for flavonoids and may also be responsible for the high activity.* Mitragyna ciliata* extracts showed moderate antiplasmodial activity towards all 3* P. falciparum* strains tested in this study, in agreement with a study conducted in 2007 where IC_50_ values ranged between 10 and 44 *μ*g/mL for various solvent extracts [39]. On the contrary, another study reported antiplasmodial activities with IC_50_ values above 100 *μ*g/mL [40]. The similarity in activities between this study and that of Adjetey and colleagues [39] may lie in the use of similar solvent systems in both studies for extraction. Interestingly, it was also reported that* M. ciliata* extract was able to modulate chloroquine activity by reversing resistance in a chloroquine-resistant strain [39]. The leaf extract of* Syzygium guineense* showed high antiplasmodial activity* in vitro* against W2 and CAM06. The antiplasmodial activity of* Syzygium guineense* has also been reported, but only in an* in vivo* model where 49% chemosuppression was observed upon administration of 400 mg/kg of extract to a rodent [41].

The antiplasmodial activities of 8 of the plants used in this study have been reported for the first time. These 8 plants are* Paspalum scrobiculatum, Acridocarpus alternifolius, Clappertonia ficifolia, Parinari congensis, Monanthotaxis caffra, Datura stramonium, Faurea speciosa, *and* Croton penduliflorus*.* Acridocarpus alternifolius* showed no antiplasmodial activity towards FCB and CAM06 strains of* P. falciparum* tested with IC_50_'s above 50 *μ*g/mL and showed only moderate activity towards W2 strain.* Datura stramonium* was also inactive towards W2 but showed moderate activity when tested against CAM06 and promising activity towards the chloroquine sensitive FCB strain. Although no antiplasmodial activity of* Datura stramonium* extracts exists in the literature, reports of the antiplasmodial activity of other* Datura* species exist.* Datura metel* has been reported to be active against both chloroquine-sensitive and chloroquine-resistant strains of the* Plasmodium parasite* [42, 43]. Other biological activities of* Datura stramonium* such as antioxidant and cytotoxicity however exist [44, 45].

*Parinari congensis* also showed promising antiplasmodial activity towards the chloroquine-sensitive FCB strain, marginally moderate activity towards CAM06 and inactivity towards W2 strain. Extracts of* Parinari congensis* have been shown to possess antioxidant, anti-inflammatory, and diabetic modulating properties [46–48].* Croton penduliflorus* showed promising activity towards all* P. falciparum* strains tested, whereas* Monanthotaxis caffra* showed moderate activities towards W2 and CAM06 strains but had promising activity towards the chloroquine-sensitive FCB strain. Other than tannins, the two extracts had little in common in terms of phytochemical profiles. Although no antiplasmodial activity record of* Monanthotaxis caffra* exists, it has been reported that extracts and compounds obtained from* Monanthotaxis parvifolia* exhibited good antiplasmodial activities [49, 50].* Paspalum scrobiculatum* and* Faurea speciosa* showed promising to moderate antiplasmodial activities against all 3 strains.* Paspalum scrobiculatum* was most active towards W2, whereas* Faurea speciosa* showed greater activity towards CAM06.* Clappertonia ficifolia* proved to be one of the most active extracts amongst the plants studied. IC_50_ values against all strains of parasites tested were well below 10 *μ*g/mL. In particular, high activity was observed against FCB with an IC_50_ of 4.43 *μ*g/mL.

For cytotoxicity, the extracts CC_50_ values were all above 30 *μ*g/mL. Extracts of* Syzygium guineense* and* Monanthotaxis caffra* were the most toxic to the LLC-MK2 cells, with CC_50_ values below 100 *μ*g/mL. All extracts were non-cytotoxic at the concentrations tested [17].* Clappertonia ficifolia*, which exhibited very good antiplasmodial activities, also had high SI towards all 3 strains (SI > 30), indicating that the extract is selective towards the parasites.* Paspalum scrobiculatum* and* Bidens pilosa* also showed good selectivity towards the chloroquine-resistant W2 strain.

In the acute toxicity, even though one death each was recorded on the first day in treatment groups that received extracts from* Syzygium guineense *and* Parinari congensis*, LD_50_ for these extracts are above 5000 mg/kg, as indicated in the OECD guidelines. No mortality was recorded for all other treatment groups over the 14 days of observation. The increase in weight in all the test groups during the duration of the experiment indicates absence of or low toxicity. Changes in body weight gain are a simple and sensitive index of toxicity after an animal has been exposed to a toxic constituent [28].

The* in vivo* antiplasmodial results indicated that* Bidens pilosa*, at a dosage of 400 mg/kg, possessed a chemosuppression rate of 74.73%. This agrees with an earlier report of a chemosuppression rate of 36% at 250 mg/kg, which is about half the dosage used for this study [35].* Clappertonia ficifolia *and* Paspalum scrobiculatum* also showed good chemosuppression rates. The results of the* in vitro* and* in vivo* antiplasmodial tests of* Clappertonia ficifolia *and* Paspalum scrobiculatum *extracts reveal that these extracts may contain compounds that when isolated could be useful in malaria drug discovery. The results also validate their use in traditional medicine for management of malaria.

## 5. Conclusion

The results of this study confirm the traditional use of these medicinal plants for the treatment of malaria and malaria-like symptoms. The toxicological results obtained suggest a need for a routine evaluation of medicinal plants to establish any toxicity before administration. Isolation of the active constituents from the most active plants is ongoing in our laboratories.

## Figures and Tables

**Table 1:** Plants selected for the study and their traditional uses.

Botanical name	Family	Local name	Ethnomedical use	Parts collected	Yield^*∗∗*^ (%)
*Paspalum scrobiculatum*	Poaceae	Star grass	Animal feed and substrate	Spikelets	5.7
*Bidens pilosa* ^*∗*^	Asteraceae	Nyamaradza	Stomach troubles	Leaves and twigs	6.2
*Acridocarpus alternifolius *	Malpighiaceae	Papao	Bacterial infections	Leaves	14.3
*Clappertonia ficifolia *	Triticeae	Sahomia	Liver malfunction	Leaves	6.3
*Mitragyna ciliata* ^*∗*^	Rubiaceae	Subaha akori	Fever	Leaves and twigs	7.5
*Parinari congensis*	Chrysobalanaceae	Krenku	Stomach ache and fever	Stem bark	11.1
*Monanthotaxis caffra *	Annonaceae	Ntetekon	Fever	Leaves and twigs	6.1
*Datura stramonium*	Solanaceae	Apple of Peru	Ear problem and cancer	Leaves and twigs	6.6
*Faurea speciosa *	Proteaceae	Setingo sebari	Malaria	Leaves and twigs	5.8
*Syzygium guineense* ^*∗*^	Myrtaceae	Senza	Antimicrobial and antifungi	Leaves	7.4
*Croton penduliflorus *Hutch	Euphorbiaceae	Nyamaradza	Body pains	Leaves and twigs	7.0

^*∗*^Antiplasmodial activity reports exist in literature. ^**∗∗**^Extract yield based on dry-powdered plant material.

**Table 2:** The major phytochemicals present in the extracts of the selected plants.

Plant Extract	Flavonoids	Alkaloids	Tannins	Sterols	Glycosides	Coumarins
*Paspalum scrobiculatum*	-	-	++	-	-	-
*Bidens pilosa*	++	-	++	++	+	-
*Acridocarpus alternifolius*	++	-	++	++	+	+
*Clappertonia ficifolia*	++	-	++	++	-	+
*Mitragyna ciliata*	-	-	++	++	-	-
*Parinari congensis*	++	-	++	++	+	-
*Monanthotaxis caffra*	-	-	++	-	-	-
*Datura stramonium*	++	-	++	-	+	+
*Faurea speciosa*	-	+	++	-	-	+
*Syzygium guineense*	++	-	++	-	-	+
*Croton penduliflorus *Hutch	+	-	++	-	+	-

(-) Absent; (+) present; and (++) strongly present.

**Table 3:** *In vitro* antiplasmodial activity of plant extracts.

Extract	IC_50_ (*μ*g *∕* mL)*∗*		
	FCB	W2	CAM06
*Paspalum scrobiculatum*	24.02 ± 0.68	6.61 ± 0.69	16.31 ± 16.31
*Bidens pilosa *	23.48 ± 5.21	4.60 ± 0.91	21.43 ± 5.99
*Acridocarpus alternifolius*	74.10 ± 1.86	36.47 ± 28.76	65.10 ± 7.23
*Clappertonia ficifolia*	4.43 ± 0.18	7.94 ± 1.36	6.56 ± 3.09
*Mitragyna ciliata*	22.63 ± 3.81	18.64 ±1.66	48.64 ± 2.27
*Parinari congensis*	12.50 ± 2.18	51.52 ± 2.17	45.09 ± 6.12
*Monanthotaxis caffra*	5.86 ± 2.76	18.94 ± 1.53	18.54 ± 0.89
*Datura stramonium*	13.29 ± 4.68	116.86 ± 1.20	46.09 ± 4.90
*Faurea speciosa*	14.83 ± 1.89	9.31 ± 1.02	6.95 ± 2.05
*Syzygium guineense*	14.94 ± 1.89	4.62 ± 1.14	5.54 ± 1.05
*Croton penduliflorus *Hutch	5.37 ± 0.18	14.03 ± 17.04	14.66 ± 2.02
Quinine	0.09 ± 0.005	0.12 ± 0.03	0.10 ± 0.05

**∗**Concentration of extract that kills 50% of *Plasmodium falciparum.*

According to [16], high (IC_50_ < 5*μ*g/mL), promising (5 < IC_50_ < 15*μ*g/mL), moderate (15 < IC_50_ < 50*μ*g/mL), and inactive (IC_50_ > 50*μ*g/mL).

**Table 4:** Cytotoxicity of crude extracts in LLC-MK2 cells and selectivity index (SI) values in the three tested *Plasmodium falciparum* strains.

Extract	CC_50_ on LLC-MK2 (*μ*g/mL)	Selectivity Index (SI)		
		FCB	W2	CAM06
*Paspalum scrobiculatum*	157.1 ± 0.98	6.54	23.77	9.63
*Bidens pilosa *	102.2 ± 0.86	4.34	22.17	4.76
*Acridocarpus alternifolius*	> 1,000	23.74	48.23	27.02
*Clappertonia ficifolia*	273.5 ± 2.02	61.74	34.45	41.69
*Mitragyna ciliata*	262.7 ± 1.66	11.61	14.09	5.40
*Parinari congensis*	150.6 ± 1.27	12.05	2.92	3.34
*Monanthotaxis caffra*	88.6 ± 0.46	15.12	4.68	4.78
*Datura stramonium*	> 1,000	128.65	14.63	37.09
*Faurea speciosa*	154.9 ± 7.89	10.45	16.64	22.29
*Syzygium guineense*	77.9 ± 0.71	5.21	16.86	14.06
*Croton penduliflorus *Hutch	272.0 ± 2.02	50.65	19.39	18.55
Gleevec (Imatinib)	18.50 ± 1.21			

CC_50_, = cytotoxic concentration 50%.

Mean and standard deviation values of CC_50_ were generated from three replicate experiments.

CC_50_ < 5: highly toxic; 5 < CC_50_ <10: cytotoxic; 10 < CC_50_ < 30: moderately to weakly cytotoxic; and CC_50_ > 30: noncytotoxic [17].

**Table 5:** Average parasitemia and percent chemosuppression of plant extracts in 4-day suppressive test.

Plant extract	% Parasitemia	% Chemosuppression
*Bidens pilosa*	7.62 ± 0.15	74.73
*Paspalum scrobiculatum*	15.24 ± 0.30	49.45
*Clappertonia ficifolia*	11.26 ± 0.23	62.64
Negative control	30.15 ± 0.24	0
Quinine	2.95 ± 0.10	90.22

## Data Availability

The data used to support the findings of this study are included within the article.
